# Clinical Relevance of Tumor Cells with Stem-Like Properties in Pediatric Brain Tumors

**DOI:** 10.1371/journal.pone.0016375

**Published:** 2011-01-28

**Authors:** Cécile Thirant, Barbara Bessette, Pascale Varlet, Stéphanie Puget, Josette Cadusseau, Silvina Dos Reis Tavares, Jeanne-Marie Studler, David Carlos Silvestre, Aurélie Susini, Chiara Villa, Catherine Miquel, Alexandra Bogeas, Anne-Laure Surena, Amélia Dias-Morais, Nadine Léonard, Françoise Pflumio, Ivan Bièche, François D. Boussin, Christian Sainte-Rose, Jacques Grill, Catherine Daumas-Duport, Hervé Chneiweiss, Marie-Pierre Junier

**Affiliations:** 1 Inserm, UMR894, Team Glial Plasticity, University Paris Descartes, Paris, France; 2 Department of Neuropathology, Hospital Sainte-Anne, Paris, France; 3 Pediatric Neurosurgical Department. Hospital Necker, University Paris Descartes, Paris, France; 4 CNRS UMR 8203, Vectorology and Anticancer Therapeutics, Gustave Roussy Cancer Institute, Villejuif, France; 5 Inserm U955, Team 10, University of Paris 12, Créteil, France; 6 Collège de France, Paris, France; 7 Laboratoire de Radiopathologie UMR 967, CEA-INSERM-Université Paris VII, Fontenay-aux-Roses, France; 8 Laboratoire d'Oncogénétique - INSERM U735, Institut Curie/Hôpital René Huguenin, St-Cloud, France; 9 Laboratoire des Cellules Souches Hématopoïétiques et Leucémiques, UMR U967, CEA-INSERM-Université Paris VII, Fontenay-aux-Roses, France; University of Bergen, Norway

## Abstract

**Background:**

Primitive brain tumors are the leading cause of cancer-related death in children. Tumor cells with stem-like properties (TSCs), thought to account for tumorigenesis and therapeutic resistance, have been isolated from high-grade gliomas in adults. Whether TSCs are a common component of pediatric brain tumors and are of clinical relevance remains to be determined.

**Methodology/Principal Findings:**

Tumor cells with self-renewal properties were isolated with cell biology techniques from a majority of 55 pediatric brain tumors samples, regardless of their histopathologies and grades of malignancy (57% of embryonal tumors, 57% of low-grade gliomas and neuro-glial tumors, 70% of ependymomas, 91% of high-grade gliomas). Most high-grade glioma-derived oncospheres (10/12) sustained long-term self-renewal akin to neural stem cells (>7 self-renewals), whereas cells with limited renewing abilities akin to neural progenitors dominated in all other tumors. Regardless of tumor entities, the young age group was associated with self-renewal properties akin to neural stem cells (P = 0.05, chi-square test). Survival analysis of the cohort showed an association between isolation of cells with long-term self-renewal abilities and a higher patient mortality rate (P = 0.013, log-rank test). Sampling of low- and high-grade glioma cultures showed that self-renewing cells forming oncospheres shared a molecular profile comprising embryonic and neural stem cell markers. Further characterization performed on subsets of high-grade gliomas and one low-grade glioma culture showed combination of this profile with mesenchymal markers, the radio-chemoresistance of the cells and the formation of aggressive tumors after intracerebral grafting.

**Conclusions/Significance:**

In brain tumors affecting adult patients, TSCs have been isolated only from high-grade gliomas. In contrast, our data show that tumor cells with stem cell-like or progenitor-like properties can be isolated from a wide range of histological sub-types and grades of pediatric brain tumors. They suggest that cellular mechanisms fueling tumor development differ between adult and pediatric brain tumors.

## Introduction

Primitive brain tumors are neoplasms of varied histopathological appearance that affect both adults and children [1], [2]. The prognosis of their most malignant forms remains poor despite development of combined therapeutic treatments. Research in cell biology and gene expression studies have provided support for the idea that tumor stem cells (TSCs), a subpopulation of cancer cells that differ from the other cancerous components by their properties similar to either neural progenitors or neural stem cells, and their peculiar resistance to current therapeutics [3]–[5], account for the origin, development and therapeutic resistance of brain tumors. Until now, TSCs have been consistently characterized only from a limited number of brain tumor types, essentially glioblastomas in adults and medulloblastomas in adults and children [6]–[13]. They form oncospheres in defined medium, which self-renew. Two types of TSCs have been isolated: TSCs that like neural stem cells have extended self-renewal properties, and TSCs that like neural progenitors or transit amplifying cells are endowed with a limited self-renewal capability. For example, TSCs isolated from pediatric and adult medulloblastomas [8], [9] differ from their counterparts isolated from adult high-grade gliomas by their limited self-renewal potential, which is closer to that of neural progenitors than to that of neural stem cells [7], [12], [14], [15]. Coherently, studies of murine medulloblastomas have shown that cerebellar progenitors are the cell of origin of these high-grade embryonal tumors [16]. TSCs share also with neural progenitors and/or neural stem cells several molecular markers [6], [8], [9], [17], and generate *in vivo* a phenocopy of the tumor from which they derive [9], [11], [17]. Experimental demonstrations that either mature glial cells having undergone a de-differentiation process [18] or that neural stem cells or neural progenitors [19], [20] may behave as the cell of origin of brain tumors suggest that most of these neoplasms could contain TSCs, regardless of their cell of origin. Independent laboratories have reported isolation of TSCs from a majority of adult high-grade brain tumors they assayed, either glioblastomas or malignant glio-neuronal tumors (MGNT) [6]-[11], [13]. Data are less numerous in children, and the different physiopathological properties exhibited by pediatric and adult brain tumors [21]–[24] limit the extrapolation of data obtained in one group of age to the other. In children, tumor cells with neural progenitor-like features have been isolated from medulloblastomas and ependymomas [25], and TSCs have been well characterized from a few cases of low- and high-grade gliomas [7], [12].

To determine the extent to which TSCs are a general component of primitive brain tumors, or are specific of certain tumor types, we performed a comparative study using a large sample of fifty-five pediatric brain tumors of various categories and grades of malignancy. The current controversies surrounding the criteria defining TSCs [11], [26]–[31], and the limited amount of data on TSCs from pediatric brain tumors led us to apply cell culture paradigms that reveal the properties of tumor cells with no *a priori* inference of the cell function from its surface markers [9], [12]. Indeed, no consensus has been achieved regarding membrane markers specific to adult glioma-derived TSCs, their expression varying among tumor samples and/or depending on environmental signals [11], [26]–[28]. We also paid special attention to the two TSC properties consistently shown throughout the extensive literature devoted to the subject, i.e. capacity of the cells to grow under the form of oncospheres and to self-renew either in a limited or extended manner.

Our data show that tumor cells with self-renewal properties and markers of neural progenitors and neural stem cells can be isolated from a majority of pediatric brain tumors regardless of their histopathological subtypes and grades of malignancy.

## Materials and Methods

### Ethics statement

The biological study was approved by the Internal Review Board of the Biological Resource Center of the Necker Sick Children Hospital in Paris. Tumor samples were obtained after signed informed consent from the parents of children who underwent surgery at Necker hospital from February 2007 to June 2009.

### Sample collections and isolation of brain tumor cells

Tumor samples were cut into 1 mm^3^ pieces and placed into either RPMI buffer for immediate processing or into freezing medium (90% serum, 10% DMSO) prior to being progressively cooled to −80°C.

Cell dissociation was performed enzymatically [7], [11] or mechanically. All of the methods employed resulted in similar cell survival rates (range, 27–95%). Fresh or frozen biopsies yielded similar rates of viable cells. Cells were plated at 5×10^4^ cells/cm^2^ in either house-made [11] or NSA-H medium (Stem Cell, France) with 10 ng/ml FGF, 20 ng/ml EGF, and 1 µg/ml Heparin [32]. Viable cell numbers varied from 2×10^5^ to 2×10^7^ in relation to the sample size. The cells were further cultured until appearance of floating cellular spheres. Half of the culture medium was renewed each week. The ability of cells forming spheres to generate novel spheres over successive passages was evaluated through seeding the individual cells at low densities (10^4^ cells/cm^2^), starting when the spheres reached a minimum 100 µm diameter as described [32]. One self-renewal was scored when the numbers of 100 µm diameter floating spheres counted under phase contrast microscopy were at least two-fold more abundant than in the previous passage. The reported limited self-renewal capacity (≤5) of tumor-initiating cells isolated from human pediatric medulloblastomas [9], [12], and the converse ability of mouse and human neural stem cells to sustain at least seven self-renewals [14], [15] (see also supplementary information on human fetal neural stem cells), led us to distinguish cells with a self-renewal ability of two to seven doublings, from those with a self-renewal ability greater than seven. Once the cultures stabilized, the spheres were routinely dissociated once a week and plated at 10^6^ cells/75 cm^2^/10 ml. Clonal properties were assessed by seeding in 96-well plates at 1–2 cells/well or in methylcellulose containing medium as previously described [14]. The characterization of human fetal neural stem cells is provided in the supplementary data and in Figure S1.

### Molecular profiling

Flow cytometry analysis of cell surface antigens CD15 and CD133 was performed as previously described [11]. Immunocytochemical and immunohistochemical procedures and image acquisition were performed as previously described [18], [33] (Table S1). Real-time quantitative RT-PCR (qPCR) was performed as previously described [34], with each sample normalized on the basis of its content in transcripts of the TATA box-binding protein, *TBP*. Results of target gene expression are presented as Ntarget values (N*target*  =  2^Ct *sample* - Ct *TBP*^, Ct cycle threshold), which were subsequently further normalized such that the mean of the cortectomies (CX) N*target* values was 1. The nucleotide sequences of primers for *TBP* and target genes are available upon request.

### CGH array, and mutation analyses of p53

The karyotype of the isolated cells was analyzed at the Agilent Platform of the Cancer research institute of Lille (University of Lille 2, IFR 114, France) using Agilent 4X180K human CGH microarrays and by Integragen society (France) using human cytoSNP-12 microarrays from Illumina. The microarray data related to this paper have been submitted to the Array Express data repository at the European Bioinformatics institute (http://www.ebi.ac.uk/arrayexpress/) under the accession numbers E-MEXP-2909 and E-MEXP-2910 and are MIAME compliant. DNA and RNA were extracted from pediatric gliomas cultures with DNAeasy and RNeasy Mini Kit (Qiagen, France). DNA fragments were generated by reverse transcription (RT) of total RNA in a final volume of 25 µl using random primers and M-*MLV Reverse Transcriptase* (Promega, France). PCR was performed by using 1 µl of the RT reaction *and* Platinum*®* Taq DNA Polymerase High Fidelity (Invitrogen, France). The primers were designed to overlap *TP53* exons 4/5 (^5′^ CTGTGACTTGCACGTACTCC ^3′^) and exons 8/9 (^5′^ TTGGGCAGTGCTCGCTTAGT ^3′^). The thermocycling conditions were 5 min at 94°C for enzyme activation, followed by 35 cycles of 94°C for 30 sec, 60°C for 1 min, 68°C for 1 min. The PCR products (568 bp length) were purified and sequenced (Biofidal, France).

### Grafts

Mechanically dissociated oncospheres (1–2 10^4^ cells/µl PBS-glucose buffer) were grafted within the right striatum of 6- to 7-week-old male nude mice (Charles River, France) or 2-month-old male NOD-SCID-ILR2^−/−^ (NSG) mice (Jackson Laboratory, Bar Harbor, Maine, USA) (1.5–2 µl/graft i.e. 1.5–4 10^4^ cells/graft). The *in situ* development of the oncospheres was analyzed as previously described [18], [33].

### Response to genotoxic stress

Spheres were dissociated, seeded at 10^4^ cells/100 µl/vial, and cultured 24 hours prior to 5 Gy -radiation as previously described [18]. Cells seeded at 2×10^4^ cells/100 µl/vial were treated with 500 µM temozolomide (Interchim, AM195, France) or its vehicle DMSO. Cell viability was evaluated with the Cell Proliferation Reagent WST-1 kit following the manufacturer's instructions (Roche, France).

### Statistical analysis

Overall survival was calculated from the time of surgery to death or time of last follow-up appointment for surviving patients. Chi-square tests were performed to test the significance of clinical differences between subtypes. Kaplan-Meier analyses were performed for survival data using the log-rank test. The Cox model was used to study prognostic factors on overall survival. Univariate and multivariate analyses were performed for both the whole series and after exclusion of the low-grade tumors. All P-values were two sided, and P<0.05 was considered significant. Analyses were performed using SPSS 16.0 for Windows.

## Results

### Tumor and patient characteristics

Tumors were classified following the WHO classification [1] and the Sainte-Anne Hospital classification [35] when applicable (Table 1). Most tissue samples were obtained from newly diagnosed tumors, with the following exceptions: TP9, TP38, and TP82 (chemotherapy prior surgery) and TP45 (chemo-radiotherapy prior surgery). Cultured samples were excluded from the analysis when neuropathological examination showed widespread areas of necrosis, paucity of tumor cells, or signs of per-operating coagulation. Non-tumoral cerebral samples taken at distance from a cavernoma and from non-infiltrating tumors were used as controls. Fifty-five tumor samples were obtained from 52 patients (3 of them having been submitted twice to surgery). The median age of the cohort was 7.8 years (range, 1.2-15.5). During a median follow-up of 1.8 years (range, 0.1–3), 11 patients died. There was no association between age group (above and below median age) and histopathological subtypes. All patients with high-grade tumors received post-operative chemotherapy or radiotherapy, whereas those with low-grade tumors were treated with surgery alone.

**Table 1:** Tumor and patient characteristics.

NAME	DIAGNOSIS	AGE	SEX	LOCATION	SURGERY
**NON TUMORAL TISSUE**					
TP27	Non tumoral part of a cavernoma	13	M	Parietal lobe	Resection
TP30S	Non tumoral part of a pilocytic astrocytoma	2	M	Optical pathway	Resection
TP31	Non tumoral lesion	4	F	Brainstem	Biopsy
TP32S	Non tumoral part of a Choroid Plexus papilloma	3	M	Posterior fossa	Resection
**EMBRYONAL TUMORS**					
TP4	Medulloblastoma	9	M	Posterior fossa	Resection
TP6	Medulloblastoma	1.3	M	Posterior fossa	Resection
TP21	Medulloblastoma	6	M	Posterior fossa	Resection
TP36	Medulloblastoma	13	M	Posterior fossa	Resection
TP37	Medulloblastoma	7	M	Posterior fossa	Resection
TP38	Medulloblastoma^1^	9	F	Posterior fossa	Resection
TP22	Atypical Teratoid/Rhabdoid tumor	8	M	Parietal lobe	Resection
**LOW-GRADE GLIAL AND NEURO-GLIAL TUMORS**					
TP9	Pilocytic Astrocytoma	6	M	Intra-lateral ventricle and optical pathway	Resection
TP30T	Pilocytic Astrocytoma^1^	2	M	Optical pathway	Resection
TP34	Pilocytic Astrocytoma	2	F	Optical pathway	Resection
TP39	Pilocytic Astrocytoma	13	F	Posterior fossa	Resection
TP47	Pilocytic Astrocytoma	2	M	Posterior fossa	Resection
TP53	Pilocytic Astrocytoma	15	M	Spinal cord	Resection
TP55	Pilocytic Astrocytoma^1^	3	F	Optical pathway	Resection
TP57	Pilocytic Astrocytoma^1^	12	M	Posterior fossa	Resection
TP58	Pilocytic Astrocytoma	2	M	Optical pathway	Resection
TP73	Pilocytic Astrocytoma^1^	6	F	Posterior fossa	Resection
TP78	Pilocytic Astrocytoma	5	F	Hypothalamus	Resection
TP79	Pilocytic Astrocytoma	1.5	M	Posterior fossa	Resection
TP81	Pilocytic Astrocytoma	14	M	Intraventricular nodule	Resection
TP10	Ganglioglioma	1.8	M	Hypothalamus	Resection
TP12	Ganglioglioma^1^	5	F	Cerebellar peduncle	Resection
TP14	Ganglioglioma^1^	13	F	Temporal lobe	Resection
TP28	Ganglioglioma	9	M	Frontal lobe	Resection
TP35	Ganglioglioma	12	F	Posterior fossa	Resection
TP43	Ganglioglioma anaplasic	13	M	Parieto-occipital lobe	Resection
TP60	Ganglioglioma	13	M	Posterior fossa	Resection
TP68	Ganglioglioma	7	M	Cerebellar peduncle	Resection
TP77	Ganglioglioma or Dysembryoplasic neuroepithelial tumor	11	M	Temporal lobe	Resection
TP2	Angiocentric NeuroEpithelial Tumor (Oligodendroglioma A)	15	M	Temporal lobe	Resection
TP11	Angiocentric NeuroEpithelial Tumor	2	M	Frontal lobe	Resection
TP54	Fibrillary Astrocytoma II (Oligodendroglioma A)	9	M	Brainstem	Biopsy
TP17	Oligoastrocytoma II (Oligodendroglioma A)	6	F	Brainstem	Biopsy
**EPENDYMOMA**					
TP8	Ependymoma III	9	M	Frontal lobe	Resection
TP16	Ependymoma III	5	M	Frontal lobe	Resection
TP20	Ependymoma III	7	M	Posterior fossa	Resection
TP45	Ependymoma III	8	M	Cerebellar peduncle	Resection
TP48	Ependymoma III	13	M	Posterior fossa	Resection
TP56	Ependymoma III	2	F	Posterior fossa	Resection
TP62	Ependymoma III	2	M	Parietal lobe	Resection
TP64	Ependymoma III	2	M	Parieto-occipital lobe	Resection
TP65	Ependymoma III	11	F	Posterior fossa	Resection
TP76	Ependymoma II	10	F	Posterior fossa	Resection
**HIGH-GRADE GLIAL TUMORS**					
TP13	Astrocytoma III (Oligoastrocytoma A)	10	M	Brainstem	Biopsy
TP59	Astrocytoma III (Oligoastrocytoma B)	13	M	Thalamus	Resection
TP80	Astrocytoma III (Infiltrating glioma)	5	F	Brainstem	Biopsy
TP7	Oligoastrocytoma III (MGNT)	8	M	Thalamus	Resection
TP25	Oligoastrocytoma III^1^ (MGNT)	8	M	Thalamus	Resection
TP44	Oligoastrocytoma III (MGNT)	10	M	Brainstem	Biopsy
TP83	Oligoastrocytoma III (Infiltrating glioma)	5	F	Brainstem	Biopsy
TP84	Oligoastrocytoma III (Oligoastrocytoma B)	9	M	Brainstem	Biopsy
TP52	Oligodendroglioma III^1^ (Oligodendroglioma B)	13	F	Intraventricular nodule	Resection
TP15	Glioblastoma (MGNT)	5	F	Brainstem	Biopsy
TP82	Glioblastoma^1^ (MGNT)	8	M	Thalamus	Resection
TP26	Unclassifiable (MGNT)	1.2	M	Temporal lobe	Resection

The WHO [1] and, when applicable, the Sainte-Anne Hospital classification [35] in parenthesis are indicated.

1 Recurrence.

### Self-renewal abilities distinguish progenitor-like and stem-like tumor cells

**Figure 1:**
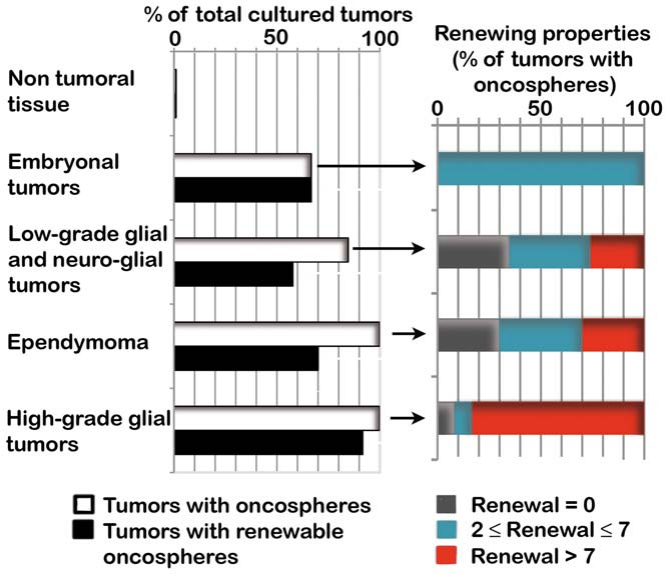
Self-renewal properties of cell-forming oncospheres derived from pediatric brain tumors. Left panel. Percent of tumors yielding cell-forming oncospheres (white bars) and oncospheres able to self-renew at least twice (dark bars). **Right panel**. The majority of high-grade glioma-derived oncospheres exhibited higher self-renewal properties than oncospheres derived from embryonal tumors, low-grade glial and neuro-glial tumors or ependymomas. Grey bars: cultures containing oncospheres devoid of self-renewal properties. Blue bars: cultures containing oncospheres with limited self-renewal abilities akin to neural progenitor-like cells. Red bars: cultures containing oncospheres with extended self-renewal properties akin to neural stem-like cells.

Cells derived from non-tumoral tissues did not form floating spheres and did not survive beyond 1.5 months. In contrast, floating cellular spheres (hence after designed as oncospheres) were observed in 49/55 tumor cultures (Figure 1 and Figure S2). Only part of the cultures containing oncospheres exhibited self-renewal capabilities (Figure 1, left panel). Twelve of the 55 tumors yielded cells forming spheres that upon dissociation either did not generate novel spheres or generated spheres in numbers smaller than or similar to the ones evaluated in the previous passage. In those cases, sphere formation was likely to result from cell aggregation. Such a situation was encountered for both low and high malignant tumors (Figure S2). Importantly, cells forming spheres without self-renewal properties did not form tumors *in vivo* (see below).

Self-renewal properties were then assessed through evaluating for each individual culture the numbers of secondary spheres generated by dissociated cells at each passage. One self-renewal was scored when the numbers of secondary spheres doubled, as compared to the previous passage. Cells with limited self-renewal properties yielded secondary spheres at least twice but less than 7 times. Cells with extended self-renewal properties exhibited at least 7 self-renewals (see the Material and Methods section). We observed a strong association between the type of self-renewal properties (either limited like neural progenitors or extended like neural stem cells) and the histopathological subtypes (P = 0.002, chi-square test, Figure 1, right panel). All medulloblastoma-derived oncospheres exhibited the limited self-renewal abilities of progenitor-like cells (Figure 1 and Figure S3). Cells with progenitor-like self-renewing properties dominated in low-grade gliomas and neuro-glial tumors, whereas high-grade gliomas yielded essentially cells with extended self-renewal properties (Figure 1 and Figure S3). Among these tumor types, isolation of cells with self-renewal properties akin to neural stem cells was associated with malignant status (P = 0.014, chi-square test, Figure 1). Regardless of tumor entities, the young age group was associated with self-renewal properties akin to neural stem cells (P = 0.05, chi-square test).

In physiological situations, only neural stem cells and, to a lesser extent, neural progenitors form colonies in a semi-solid medium or when cultured under limiting dilutions [14]. Clonal cell frequencies were assayed using these assays on nine cultures of pediatric tumors. They ranged from 0.07% (for a ganglioglioma culture containing floating spheres that did not self-renew) to 86% (for an oligoastrocytoma III culture) (Figure S4). This wide range of frequency of oncosphere-forming cells was similar to those reported in previous studies of adult brain tumor TSCs [7], [9], [25].

High-resolution oligonucleotide array-CGH analysis showed that cells forming spheres exhibited severe chromosomal aberrations in each of the cultures examined (Figure S5). In addition, we identified distinct point mutations of the *TP53* gene in oncospheres derived from four tumor samples: TP54 (R248Q), TP59 (R273C), TP80 (R273H), and TP84 (R158G). Survey of p53 nuclear immunoreactive signal accumulation, a *TP53* mutation surrogate [36], was performed in a subset of the original tumor panel using the surgical samples preserved for histo-pathological analysis. The results showed ≥50% p53-immunoreactive cells in 7/10 high-grade gliomas (Table S2), in accordance with reported frequencies of *TP53* mutations in pediatric malignant gliomas [37].

In summary, cells demonstrating ability to form self-renewing oncospheres were obtained in 37 of the 55 pediatric brain tumors studied. They were observed in the majority of tumors within each broad histopathological and grade categories studied. All cultures containing cells with limited self-renewal properties stopped proliferating within 5 months on average. Of note, only 30% of the cultures containing cells with extended self-renewal capacities (6/19), all derived from grade II-IV gliomas (one astrocytoma II, two astrocytoma III, two oligoastrocytoma III and one glioblastoma), could be maintained beyond 6 months of culture. The others underwent a proliferation arrest, followed by their progressive disappearance.

### Tumor cells with prolonged self-renewal properties are associated with poor patient outcome

**Figure 2:**
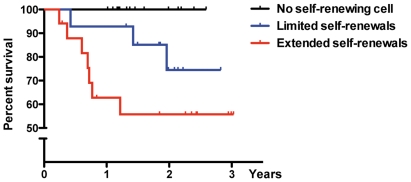
Association of self-renewal capacities with survival. Kaplan-Meier curves of the whole cohort show that patients whose tumor had led to cells having self-renewal properties akin to neural stem cells had a poorer prognosis compared to those with progenitor-like properties or those devoid of renewal abilities (P = 0.022, log-rank test). Crosses placed over the curves mark the time of the loss of a patient (death or last appointment follow-up).

We evaluated the prognostic factor of patients with self-renewing tumor cells. For the whole cohort (45 patients for which clinical information was available) we found a strong statistical correlation between the dead/alive status of the patient and the self-renewal properties of the tumor cells (P = 0.001, chi-square test), tumor cells with limited neural progenitor-like self-renewal properties defining a group of patients with intermediate survival expectancies (Figure 2). At the time of analysis (November 2010), only 55% of the patients with a tumor containing “stem-like tumor cells” were alive, versus 78% and 100% with tumors containing “progenitor-like tumor cells” and no self-renewing cells, respectively. Survival analysis of the whole cohort indicated that tumors containing “stem-like tumor cells” were associated with a poorer outcome (P = 0.013, log-rank test; Figure 2). Although patients with tumors containing “progenitor-like cells” appeared to constitute an intermediate group of survival, this group did not reach statistical significance as compared with either of the other groups. Multivariate Cox regression analysis in the full cohort showed that isolation of cancer cells with extended self-renewal properties from the tumors of the patients were a significant prognostic marker for survival, independent of age group and histopathological subtype (95% CI, 0.07 to 0.79; P = 0.018). However, no statistical significance was reached when the analysis was restricted to the high-grade gliomas subgroup.

### Neural stem cell and mesenchymal molecular profiles in pediatric brain tumor-derived oncospheres

**Figure 3:**
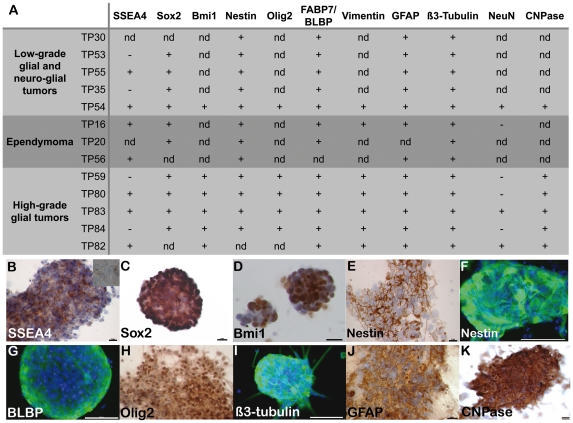
Self-renewing cell-forming spheres derived from both low-grade and high-grade gliomas have a neural stem cell profile combined with neural progenitor markers indicative of both pro-neuronal and pro-glial potencies. **A.** Summary of the immunocytochemical analysis of glioma-derived cells forming self-renewing oncospheres. nd: not determined. +: immunoreactive cells. -: no immunoreactive cells. See text for further details. **B-K.** Examples of immunoreactive cells. **B.** SSEA4-immunoreactive cells in floating spheres derived from an astrocytoma II. **C.** Sox2-immunoreactive cells derived from an oligoastrocytoma III. **D.** Bmi1-immunoreactive cells derived from an astrocytoma III. **E.** Nestin-immunoreactive cells derived from an astrocytoma II. **F.** Nestin-immunoreactive cells derived from an ependymoma. **G.** BLBP/FABP7-immunoreactive cells derived from an ependymoma III. **H.** Olig2-immunoreactive cells derived from an astrocytoma III. **I.** β3-tubulin-immunoreactive cell-forming spheres derived from a pilocytic astrocytoma **J.** GFAP-immunoreactive cells derived from an oligoastrocytoma III. **K.** Example of CNPase-immunoreactive cells in an astrocytoma II culture. Bar = 15 µm in B and K, 20 µm in C, H and J; 25 µm in D, E; 100 µm in F, G, and I. In panels E, H, J and K, the floating oncospheres were smeared onto a glass slide prior to being submitted to the immunocytochemical procedure. B-K, Hemalun or DAPI counterstaining.

Pediatric glioma self-renewing cells exhibited a neural profile. In all cultures studied by immunocytochemistry, cell-forming spheres shared markers with human fetal neural stem cells (Figure 3). Immunocytochemistry showed that Sox2 and Bmi1, which are necessary for the normal functioning of both embryonic stem cells and neural stem cells [39], [40], were expressed by a majority of cells in the low- and high-grade gliomas cultures studied (10/10 and 6/6 cultures, respectively, Figure 3A, C, and D). Likewise, the neural stem cell marker Nestin was detected in the majority of the cells in all cultures examined (12/12 cultures, Figure 3A, E, and F). In addition, we observed cells immunoreactive for the stage-specific embryonic antigen 4 (SSEA4) a marker of embryonic stem cells and of the early neuro-epithelium [38] in seven of the ten cultures we assayed and that were derived from low- and high-grade glial tumors (Figure 3A–B). In accordance with the well-known co-existence of stem cells with their progeny at different stages of differentiation in neural stem cell as well as TSC cultures growing under the form of floating spheres [6], [41], we observed cells immunoreactive for the neural progenitor markers BLBP/FABP7 and Olig2 in oncospheres of the glial tumors examined (12/12 and 5/5 cultures, respectively, Figure 3A, G, H). Immunocytochemistry additionally showed a widespread and robust expression of Vimentin (Figure 3A and Figure S6a), an intermediate filament that, like Nestin, is expressed by neural stem cells and neural progenitors in the developing CNS. Of the markers of neuronal lineage, only β3-tubulin, which is normally restricted to neuroblasts and immature neurons in the normal CNS and is known to be expressed by tumoral glial cells in adults [47], was observed in all cultures examined (20 to 50% of positive cells in 13/13 cultures, Figure 3A, and I). Conversely, cells expressing NeuN, a marker of mature neurons, were exceptionally observed (rare cells in 3/7 cultures, Figure 3A and Figure S6b). Cells expressing GFAP, which is normally observed *in vivo* in astrocytes and in neural progenitors and neural stem cells of the developing human brain [48], were abundant (>50% of the cells) in all our cultures (12/12 cultures, Figure 3A, and J). Surprisingly, the enzyme CNPase that is normally produced by myelinating oligodendrocytes, was expressed by the majority of cells in the six cultures examined (Figure 3A, and K).

**Figure 4:**
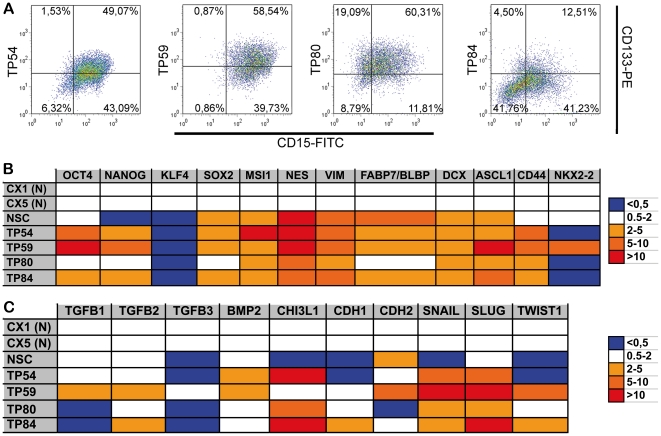
Neural stem cell and mesenchymal molecular profiles of pediatric brain tumor-derived oncospheres. **A**. Fluorocytometric analysis of the expression of the neural stem cell markers CD15 and CD133 by cells derived from low- (TP54) and high-grade gliomas (TP59, 80, and 84). **B**. Comparative qPCR analysis of cell lineage marker expression in different pediatric glioma-derived cells (TP) and human fetal neural stem cells (NSC). Results are presented relative to the mean transcript levels measured in controls (adult human biopsies of cortices from epileptic patients, CX1, CX5). Astrocytoma II-derived oncospheres (TP54), astrocytoma III-derived oncospheres (TP59, TP80), oligoastrocytoma III-derived oncospheres (TP84). Musashi 1 (MSI1), Nestin (NES), Vimentin (VIM), brain lipid binding protein (FABP7/BLBP), Doublecortin (DCX). **C**. Altered transcript levels of several genes normally expressed in mesenchymal cells distinguish pediatric glioma-derived cultures (TP) from neural stem cells (NSC).

We completed this study by determining in one low-grade and three high-grade glioma-derived cultures the expression of the neural stem cell markers Prominin/CD133 and SSEA1/CD15 as well as the levels of embryonal/neural stem cells and mesenchymal markers as compared to neural stem cells. FACS revealed that a majority of the cells expressed Prominin/CD133, SSEA1/CD15, or both proteins in the four cultures (Figure 4A). Comparative profiling of tumor-derived oncospheres and neural stem cells with regards to markers of embryonic stem cells, neural stem cells, neural progenitors, and mesenchymal cells was achieved using qPCR. A mesenchymal signature has recently been shown to be associated with the expression of several markers of neural stem cells in adult glioblastomas TSCs [49]. Transcripts of the embryonic stem cell markers Oct4, Nanog, and - at low levels - Klf4 were detected in the four tumor cultures examined (one astrocytoma II, two astrocytoma III and one oligoastrocytoma III that yielded cell-forming spheres with extended self-renewal properties, Figure 4B). Surprisingly, three of them exhibited enhanced Oct4 and Nanog transcript levels as compared to both control tissues and fetal neural stem cells (Figure 4B). Enhanced levels of transcripts encoding Sox2, Musashi 1, Nestin and Vimentin were observed in the glioma cultures assayed and in neural stem cells as compared to adult cortical tissues (Figure 4B). We also observed expression of transcripts encoding the neural progenitor marker BLBP/FABP7 in neural stem cells and in glioma-derived cell spheres (Figure 4B). Enhanced levels of Achaete-scute complex Homolog 1 (ASCL1) and Doublecortin (DCX) transcripts, markers of neural progenitors engaged in the early steps of neuronal differentiation [42], [43], were observed in human fetal neural stem cells and all glioma cell cultures examined as compared to adult cortical tissues (Figure 4B). In contrast, increased transcript levels of hyaluronan/CD44, which is normally expressed by astrocyte progenitors [44] and frequently overexpressed in TSCs derived from solid tumors (including gliomas) [45], were observed in all the tumor cultures assayed but not in the human fetal neural stem cells, as compared to adult cortical tissues (Figure 4B). Conversely, NKX2.2 expression, which is normally present in neural progenitors engaged in the early steps of oligodendroglial differentiation [46], was down-regulated except in TP59, one of the two astrocytoma III cultures included in this assay (Figure 4B). We finally observed enhanced transcript levels of several genes known to play crucial roles in the adoption of a mesenchymal phenotype [50] in all the cultures we tested, as compared to both control tissues and fetal neural stem cells (Figure 4C).

Taken together, these results show that despite an extensive diversity of molecular markers, cell-forming spheres derived from both low-grade and high-grade gliomas have a common neural stem cell profile combined with neural progenitor markers indicative of both pro-neuronal and pro-glial potencies. This molecular profile comprises elements known to control mesenchymal transitions in the low- and high-grade glioma cultures we sampled.

### In vivo formation of highly infiltrative tumors by pediatric glioma-derived oncospheres

**Figure 5:**
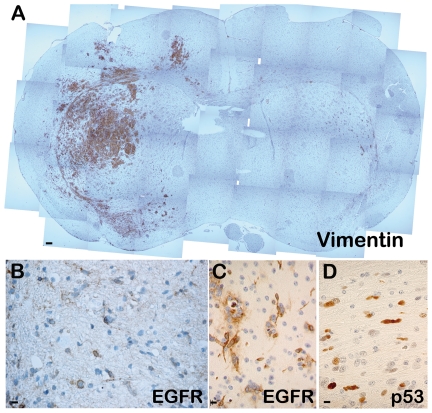
Tumor formation by cells with stem-like properties derived from pediatric gliomas. **A**. Example of tumor formation by cells derived from an astrocytoma II culture 6 months after grafting into the right striatum of nude mice. The grafted cells were identified using an antibody specific to the human form of Vimentin. As in the original patient tumor (**B**), the cells that composed the mouse tumors expressed EGFR (**C**) and exhibited p53-immunolabeling (**D**). Hemalun counterstaining. Bars = 110 µm in A, 10 µm in B, C and D.

**Figure 6:**
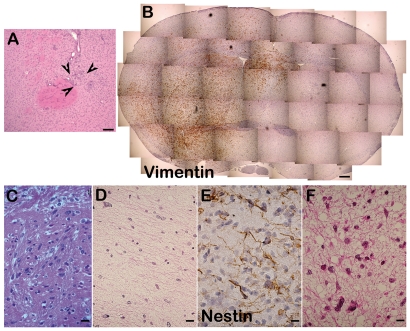
Tumor initiating properties of glioma-derived oncospheres with and without self-renewal abilities. **A**. Grafting of cell forming spheres derived from a ganglioglioma (TP77), which lacked self-renewal ability resulted in small accumulation of cells (arrows) but not in tumor formation. **B–D**. *In vivo* tumor formation by tumor cells with stem-like properties derived from pediatric gliomas. **B**. Two out of five mice grafted with cells derived from an astrocytoma III (TP80) exhibited a robust development of tumor cells, which reproduced the appearance of the patient original tumor, as observed using Vimentin-immunolabeling. **C–D**. The histological aspect of the mouse tumor (**C**) was comparable to that of the patient original tumor (**D**). **E–F**. A large, infiltrating tumor was observed 6 months after grafting into the right striatum in one of the three NSG mice grafted with glioblastoma-derived cells (TP82). Immunolabeling with an antibody that recognized the human but not the rodent form of Nestin (**E**) revealed cells endowed with an elongated, bipolar morphology similar to that observed in the patient tumor (**F**). C and F, hemalun/phloxin counterstaining. A, B, D, E, hemalun counterstaining. Bar = 100 µm in A, 250 µm in B, 20 µm in C and D, 10 µm in E and F.

To determine their tumor-initiating properties, cell-forming spheres with self-renewing ability derived from three cultures (TP54, TP80, TP82) were grafted into the brains of nude or NSG mice, which were sacrificed 3.5 to 6 months post-graft. In the three mice grafted with TP54-derived cells, tumor cells were observed throughout both hemispheres (Figure 5A). As in the original patient tumor (an infiltrating brainstem glioma), numerous cells were disseminated, were EGFR-immunoreactive (compare Figure 5B with 5C), and had abnormal nuclei with p53-immunoreactivity (Figure 5D and E). Similarly, grafts of TP80- and TP82-derived cells resulted in tumor formation (Figure 6). Among the cultures containing oncospheres that did not self-renew, one (TP77) yielded a numbers of spheres sufficient to allow determining their tumor-initiating properties *in vivo*. Grafting of TP77-derived oncospheres in the brain of immunodeficient mice resulted only in a modest cell accumulation at the injection site (Figure 6A), showing that these cells devoid of self-renewal properties *in vitro* lacked also tumor-initiating properties *in vivo*.

Together, these results indicate that the tumor cells with long-term self-renewing capacities we engrafted in mouse brains resulted in the *in vivo* development of tumors that recapitulated the histological and molecular profile of the original human tumor.

### Pediatric glioma-derived oncospheres are resistant to genotoxic stress

**Figure 7:**
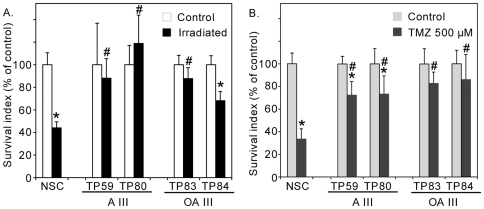
Resistance of pediatric high-grade glioma-derived tumor stem-like cells to irradiation (A) and temozolomide (B) compared to human fetal neural stem cells (NSC). A: astrocytoma. OA: oligoastrocytoma. Data represent the mean ± s.d. (n = 3). Student-Newman-Keuls test, *: p<0.001 as compared to the respective controls. #: p<0.001 as compared to irradiated NSC in (**A**) or temozolomide-treated NSC in (**B**).

According to the TSC hypothesis, tumor recurrence results from TSC resistance to current therapeutics. The radio- and chemosensitivity of cell-forming spheres derived from pediatric gliomas was evaluated using irradiation and the DNA-alkylating agent temozolomide on four glioma cultures of TSC with long-term self renewing capacities (two astrocytomas III, TP59 and TP80, and two oligoastrocytomas III, TP83 and TP84) and on human fetal brain-derived neural stem cells used for comparison. Metabolic activity, taken as a survival index, was evaluated one week post-irradiation or after exposure to a high concentration of temozolomide. As depicted in Figure 7, TSC derived from pediatric gliomas survived significantly better than neural stem cells after both treatments (Student-Newman-Keuls test, p<0.001).

## Discussion

This study provides evidence that tumor cells expressing markers and self-renewal properties of neural progenitors or neural stem cells can be isolated, and cultured from most pediatric brain tumors regardless of subtype or grade of malignancy. Further analysis of one low-grade and three high-grade glioma cultures showed that the cells we isolated expressed a complex molecular profile that combined mesenchymal and neural elements, suggesting a high potential of plasticity. Testing *in vivo* tumorigenicity, we verified that non self-renewing cells did not form tumors when engrafted in immunodeficient mice brains whereas the three long-term self-renewing ones we tested generated tumors respecting the histological and molecular profile of the original human lesion. Of note, as their adult counterparts [4], [5], the four children gliomas-derived TSC tested here resisted to chemo- or radiotherapy.

Although cell-forming self-renewing spheres isolated in this study did not share a unique molecular signature, they all presented a molecular profile highly similar to that expected for neural progenitors and/or neural stem cells [40]. This immature neural profile was associated in the four TSCs cultures we tested with high-transcript levels of molecules normally present in mesenchymal cells including CHI3L1/YKL40, which is also known as a marker of adult glioblastoma aggressiveness [51]. This observation echoes those recently reported in adult glioma tissues [52] and in adult glioblastoma-derived TSCs [49], [53], and lends credence to the view that brain TSCs could be characterized by the unique association of a neural stem cell profile and of a mesenchymal profile essential for maintaining the tumor initiating properties of the cells [49]. Epithelial to mesenchymal transition is a well-known event, which presides on epithelial cancer metastasis [50], and the most aggressive forms of adult gliomas cluster with a mesenchymal signature [52]. Most importantly, sharing of a common molecular profile by cells obtained from pediatric and adult tissues, and from different gliomas subtypes with different genomic alterations reinforces the idea that glioma cancer cells with stem properties play crucial roles in glioma development.

Our observation that tumor cells with self-renewal properties and markers of neural progenitors and neural stem cells can be isolated from a majority of pediatric brain tumors favors our initial hypothesis that TSCs are a common component of brain tumors. Our success in derivation of self-renewing oncospheres from pediatric high-grade brain tumors is comparable with or above the 50% average success rate reported to date on adult high-grade gliomas using the neurosphere assay [7], [9], [11], [13], [54], [55]. During the redaction of the present work, a study of 56 pediatric tumors described the obtaining of oncospheres passageable three times from 50% high-grade glial tumors (high-grade gliomas and anaplasic ependymomas), and 20% pilocytic astrocytomas [56], compared to 85% and 61% observed here for pediatric tumors of the same histological sub-types. Lack of self-renewing oncospheres in a minority of the surgical samples cultures may result from either absence of such cell types in the tumors or from their requirement of specific growth conditions. The higher yield of self-renewing oncospheres we observed within a given tumor category, when compared to Panoysan and colleagues [56], was especially marked for low-grade neoplasms. This observation suggests that the ability to derive cells with properties akin to neural progenitors or neural stem cells from a given tumor could be more dependent on the absolute numbers of cells endowed with progenitor or stem properties within the cultured tumor sample, than on the optimization of culture conditions for each histological tumor sub-type. Further experiments are necessary to address this issue.

Our analysis over the whole tumor cohort showed a correlated segregation into three distinct sub-groups between survival expectancy and the type of self-renewing properties of the cancer cells, independent of age group and histopathological type of the tumor. The predictability of this correlation for individual patients must be moderated according to the finding of tumor cells with limited or extended self-renewing properties in low-grade tumors from patients that remained clinically stable without any post-surgical treatment, and the fact that we obtained such cells from a large majority of tumors within all categories examined. However, distinction of an intermediate survival group formed by tumor cells with limited self-renewal properties indicates that the degree of immaturity of these cells may provide a pertinent novel model for the stratification of pediatric brain tumor prognosis. The most intriguing result of our study is the finding of tumor cells with properties akin to neural progenitors or neural stem cells in most of the tumors examined. Although the frequency of TSCs derivation was higher in high-grade pediatric tumor, our data indicate that in children such cells were not the monopoly of the most malignant form of glial tumors contrary to what can be inferred from the studies on adult brain tumors [6]–[11], [13]. This could suggest that cells with stem cell-like or progenitor-like properties are more frequent components of pediatric brain tumors than of adult brain tumors. Consequently, TSCs could be an essential driving force of pediatric brain tumors and a dispensable element of adult ones. Alternatively, the fact that such cells were not systematically found in all tumors within a given category, opens the possibility that they are not present at all time-points of the tumor development. The exceptional plasticity of normal somatic cells, exemplified by the ability of glial progenitors [57], and the ability of mature glial cells [58], [59] to be converted into stem-like cells in response to defined environmental conditions could suggest that brain cancer cells can acquire properties of progenitor cells or of stem cells in response to permissive environmental pressure. Such a possibility would imply that therapeutic anti-cancer strategies should take cell plasticity into account.

## Supporting Information

Figure S1 **Isolation and characterization of neural stem cells from human fetal brain.** All studies with human tissue were performed under Ethical Approval from the University Paris-Descartes internal review board using tissue donated with informed consent after elective termination of pregnancy. Human fetal brain at embryonic day 50-55 (Carnegie stage 19-22) were carefully dissected and mechanically dissociated into single cell suspensions. Primary cells were cultured under the form of floating spheres in NeuroCult® NSC Basal Medium supplemented with NeuroCult® Proliferation Supplements (StemCell Technologies), human EGF 20 ng/mL, human b-FGF 10 ng/mL (AbCys) and Heparin 20 ng/mL (Sigma-Aldrich) final. Two primary cultures of neural stem cells (NSC24 and NSC25) were established from two distinct fetuses, and expanded with a passage every two weeks. No culture crisis, spontaneous differentiation or abnormal genetic derivation as assessed by CGH array was detected over one year of continuous culture. Clonal properties were determined after 4 months of culture (passage 12) by manual deposition of 1 cell/100 µL into non-coated 96-well plates. Fifty µL of medium was added every two weeks during two months. 5.9%±1.2 of CSN24 cells, and 4.5%±1.6 of CSN25 cells yielded spheres in these conditions. Immunocytochemical analysis showed that most cells expressed the NSC markers Sox2, Bmi1 and Nestin, while no cell immunoreactive for Synaptophysin, phosphorylated neurofilaments, and NeuN was observed. **Left panel**: Immunocytochemical labeling showing protein expression of the NSC markers Bmi-1, Sox2 (brown nuclei staining) and Nestin (brown cytoplasm staining). Hemalun counterstaining. Scale bar = 25 µm. **Right panel**: Entire genome profile of NSC using HumanCytoSNP-12 Beadship. These graphs represent the allelic copy ratio in terms of B allele frequency (diagnosing genetic aberrations) and the log2 ratio of the fluorescence intensities along each chromosome noted log R ratio (monitoring physical aberrations). Copy number was normal (CN = 2), no duplication or deletion were detected.
(TIF)

Figure S2 **Distinct behavior in serum-free medium cultures of cells derived from low-grade gliomas and neuro-glial tumors as compared with other pediatric brain tumors-derived cells. a.** As expected, cells growing under the form of floating oncospheres were observed in cultures of medulloblastoma (MDB). **b–n**. Oncospheres were observed in cultures derived from both low- and high-grade brain tumors. Cultures of pilocytic astrocytomas and gangliogliomas were characterized with anchored clusters of round shaped cells from which cells with long extensions departed. Floating cellular spheres budded from the attached cell networks. Over time, cells could spread between the clusters, forming loosely dense networks. Only the floating spheres could be expanded. All attempts to amplify the cell networks through re-seeding were unsuccessful. **b–c**. Cultures of pilocytic astrocytomas formed cell networks anchored to the culture vials (**b**) prior to develop under the form of oncospheres (**c**) that coexisted with anchored cells. **d–e**. Ganglioglioma derived cells formed anchored cell networks developing from cellular aggregates (**d**) prior to yield oncospheres (**e**). Among the low-grade gliomas cultured, only the grade II astrocytoma-derived cells developed straightforwardly under the form of floating spheres. **f–g**. All ependymoma cultures contained floating spheres. Ependymoma-derived cells exhibited various growth aspects from one sample to another. They grew either under the form of anchored cells prior to yield oncospheres (**f**) or directly under the form of free-floating oncospheres (**g**). **h–n.** Floating spheres were observed in all high-grade gliomas cultures within two weeks post-seeding, and dominated over anchored cells. **h–j.** Cells derived from astrocytomas grew under the form of oncospheres (**h** and **j**) coexisting with cells and cellular aggregates loosely anchored to the culture vial (**i**). **k.** Oligoastrocytoma-derived cells developed as oncospheres. **l–m.** Glioblastoma-derived cells developed under the form of patches of anchored-cells, which yielded oncospheres. **n.** Example of oncospheres derived from a tumor corresponding to a MGNT according to Sainte-Anne hospital classification.
(TIF)

Figure S3 **Self-renewal properties of oncospheres derived from each tumor category.** The absolute numbers of tumors are indicated on each bar. Grey bars: tumor-derived cultures containing oncospheres devoid of self-renewal properties. Blue bars: tumor-derived cultures containing oncospheres with limited self-renewal (SR) ability akin to neural progenitor-like cells (2≤SR≤7). Red bars: tumor-derived cultures containing oncospheres with extended self-renewal property akin to neural stem-like cells (SR>7). In **a**, the tumors are categorized along the WHO classification and in **b** along the Sainte-Anne hospital classification.
(TIF)

Figure S4 **Clonal properties of cell-forming spheres** (a). **b-c.** Example of colony obtained in methylcellulose medium for a ganglioglioma culture (**b**) and an astrocytoma culture (**c**).
(TIF)

Figure S5 **Examples of cytogenomic profiles of oncospheres derived from a paediatric astrocytoma II (a) and III (b).** Aberrations were obtained with the ADM2 algorithm and filtering options of a minimum of 3 probes and abs (log 2 ratio) = 0.4. Profiles of segmental chromosomal copy number alterations (log 2 ratio>0.4) are represented with green (losses) and red (gains) lines.
(TIF)

Figure S6 **Examples of vimentin, and neuN expression in oncospheres derived from an oligoastrocytoma III (a) and a fibrillary astrocytoma II (b).** Bar = 20 µm in a, 10 µm in b.
(TIF)

Table S1 **Antibodies used for immunocyto- (ICC), immunohistochemical (IHC), and flow cytometry (FACS) analyses.** All incubations for ICC and IHC were performed for 1 hour at room temperature unless otherwise indicated. Cells growing under the form of cellular spheres were collected, mechanically spread onto Superfrost glass slides (Dutscher, France), and fixed for 10–30 min in ethanol at room temperature. Cells growing in an anchored manner were directly treated with 2–4% paraformaldehyde in PBS for 10 min. Eight µm-thick sections were prepared from paraffin-embedded mouse brains, and from initial tumor samples, and 30 µm-thick free-floating sections from frozen mice brains. For FACS, cells were labelled with anti CD133-PE and anti CD15-FITC antibodies in a ratio of 5 µL of each antibody per 10^6^ cells in a total volume of 100 µL for 30 minutes at 4°C. Isotype controls coupled to the same fluorophores were used as control antibodies. Data acquisition was performed on FACScalibur (BD Biosciences) and analysed using FlowJo software (Tree Star Inc.).
(DOC)

Table S2 **Immunohistochemical detection of p53 in a subset of the original patient tumor panel.**
(DOC)
