# Supplementary material for: Clinical Relevance of Tumor Cells with Stem-Like Properties in Pediatric Brain Tumors
Source: PLoS One. 2011 Jan 28;6(1):e16375. doi: 10.1371/journal.pone.0016375 (PMC3030582; doi:10.1371/journal.pone.0016375)
Supplement: Table S1 — Antibodies used for immunocyto- (ICC), immunohistochemical (IHC), and flow cytometry (FACS) analyses. All incubations for ICC and IHC were performed for 1 hour at room temperature unless otherwise indicated. Cells growing under the form of cellular spheres were collected, mechanically spread onto Superfrost glass slides (Dutscher, France), and fixed for 10–30 min in ethanol at room temperature. Cells growing in an anchored manner were directly treated with 2–4% paraformaldehyde in PBS for 10 min. Eight µm-thick sections were prepared from paraffin-embedded mouse brains, and from initial tumor samples, and 30 µm-thick free-floating sections from frozen mice brains. For FACS, cells were labelled with anti CD133-PE and anti CD15-FITC antibodies in a ratio of 5 µL of each antibody per 106 cells in a total volume of 100 µL for 30 minutes at 4°C. Isotype controls coupled to the same fluorophores were used as control antibodies. Data acquisition was performed on FACScalibur (BD Biosciences) and analysed using FlowJo software (Tree Star Inc.). (DOC) [file pone.0016375.s007.doc]

| **Antigene** | **Antibody** | **Species** | **Manufacturer** | **FACS** | **ICC** | **IHC with unmasking** |
| --- | --- | --- | --- | --- | --- | --- |
| ß3 tubulin | Monoclonal | Mouse | Covance, Eurogentec, Belgium |  | 1:3000 |  |
| CD133-PE | Monoclonal | Mouse | Miltenyi, France | 1:20 |  |  |
| CD15-FITC | Monoclonal | Mouse | BD Biosciences, France | 1:20 |  |  |
| BLBP | Polyclonal | Rabbit | Santa Cruz, TEBU, France |  | 1:100 |  |
| BMI-1 | Monoclonal | Mouse | Upstate, Millipore, France |  | 1:50, unmasking |  |
| CNPase | Monoclonal | Mouse | Abcam, France |  | 1:500 | 1:250 |
| EGFR | Monoclonal | Mouse | Ventana Medical System, France |  | Pure, protease unmasking | Pure, protease unmasking |
| GFAP | Monoclonal | Mouse | Dakocytomation, France |  | 1:1000 | 1:200 |
| GFAP | Polyclonal | Rabbit | Dakocytomation , France |  | 1:1000 | 1:500 |
| KI-67 | Monoclonal | Mouse | Dakocytomation, France |  | 1:150 | 1:75 |
| Nanog | Polyconal | Rabbit | Abcam, France |  | 1/200 |  |
| Nestin | Monoclonal | Mouse | Chemicon, Millipore, France |  | 1:500 | 1:500 |
| NeuN | Monoclonal | Mouse | Chemicon, Millipore, France |  | 1:500, unmasking | 1:500 |
| OLIG-2 | Polyclonal | Goat | R&D systems, France |  | 1:150 | 1:150 |
| p53 | Monoclonal | Mouse | Beckman Coulter, Immunotech, France |  | pure | Pure |
| SOX-2 | Monoclonal | Mouse | Neuromics, Acris, Germany |  | 1:250 | 1:50 |
| SSEA-4 | Monoclonal | Mouse | Chemicon, Millipore, France |  | 1:200 |  |
| Synaptophysin | Monoclonal | Mouse | Progen, Germany |  | 1:50, unmasking | 1:50 |
| Vimentin | Monoclonal | Mouse | Dakocytomation, France |  | 1:2000 | 1:400 |
