# Supplementary material for: Clinical Relevance of Tumor Cells with Stem-Like Properties in Pediatric Brain Tumors
Source: PLoS One. 2011 Jan 28;6(1):e16375. doi: 10.1371/journal.pone.0016375 (PMC3030582; doi:10.1371/journal.pone.0016375)
Supplement: Table S2 — Immunohistochemical detection of p53 in a subset of the original patient tumor panel. (DOC) [file pone.0016375.s008.doc]

**Table S2. Immunohistochemical detection of p53 in a subset of the original patient tumor panel.**

| **Name** | **Diagnosis** | **p53** |  | **Name** | **Diagnosis** | **p53** |
| --- | --- | --- | --- | --- | --- | --- |
|  | **Low grade glial and neuro-glial tumors** |  |  |  | **Ependymoma** |  |
| TP9 | Pilocytic Astrocytoma | 0 |  | TP8 | Ependymoma III | ≥15% |
| TP30T | Pilocytic Astrocytoma | ≥10% |  | TP16 | Ependymoma III | ≥20% |
| TP34 | Pilocytic Astrocytoma | 0 |  | TP20 | Ependymoma III | ≥5% |
| TP39 | Pilocytic Astrocytoma | 0 |  | TP45 | Ependymoma III | 0 |
| TP47 | Pilocytic Astrocytoma | 0 |  | TP48 | Ependymoma III | 0 |
| TP53 | Pilocytic Astrocytoma | 0 |  | TP56 | Ependymoma III | 0 |
| TP57 | Pilocytic Astrocytoma | 0 |  | TP62 | Ependymoma III | 0 |
| TP73 | Pilocytic Astrocytoma | 0 |  | TP64 | Ependymoma III | ≥25% |
| TP78 | Pilocytic Astrocytoma | 0 |  | TP65 | Ependymoma III | 0 |
| TP79 | Pilocytic Astrocytoma | 0 |  | TP76 | Ependymoma II | 0 |
| TP10 | Ganglioglioma | 0 |  |  | **High-grade glial tumors** |  |
| TP12 | Ganglioglioma | 0 |  | TP13 | Astrocytoma III (Oligoastrocytoma A) | ≥50% |
| TP14 | Ganglioglioma | 0 |  | TP59 | Astrocytoma III (Oligoastrocytoma B) | ≥60% |
| TP28 | Ganglioglioma | ≥20% |  | TP25 | Oligoastrocytoma III (MGNT) | ≥80% |
| TP35 | Ganglioglioma | 0 |  | TP44 | Oligoastrocytoma III (MGNT) | ≥80% |
| TP43 | Ganglioglioma anaplasic | ≥10% |  | TP83 | Oligoastrocytoma III (Infiltrating glioma) | ≥80% |
| TP60 | Ganglioglioma | ≥10% |  | TP84 | Oligoastrocytoma III (Oligoastrocytoma B) | ≥60% |
| TP68 | Ganglioglioma | 0 |  | TP52 | Oligodendroglioma III (Oligodendroglioma B) | 0 |
| TP77 | Ganglioglioma | 0 |  | TP15 | Glioblastoma (MGNT) | ≥5% |
| TP2 | ANET (Oligodendroglioma A) | 0 |  | TP82 | Glioblastoma (MGNT) | ≥80% |
| TP11 | ANET | 0 |  |  |  |  |
| TP17 | Oligoastrocytoma II (Oligodendroglioma A) | 0 |  |  |  |  |

Results are provided in percentages of immunoreactive cells. Our series adds argument to the view that mutations in *TP53* tumor suppressor gene are frequent in pediatric malignant gliomas [1-4]. Of the 21 low-grade tumors studied, only one pilocytic astrocytoma and two gangliogliomas showed more than 10-20% of immunoreactive cells, while an astrocytoma II exhibited more than 50% of cells with high immunoreactive signal. An anaplasic ganglioglioma was also found to contain 10% of immunoreactive cells. Five-25% of p53-immunoreactive cells were observed in four of the ten ependymoma surveyed, whereas 7 out of 10 high-grade gliomas exhibited 50-80% of p53-immunoractive cells, and one 5%.

References:

[1] I.F. Pollack, R.L. Hamilton, S.D. Finkelstein, J.W. Campbell, A.J. Martinez, R.N. Sherwin, M.E. Bozik, S.M. Gollin, The relationship between TP53 mutations and overexpression of p53 and prognosis in malignant gliomas of childhood, Cancer Res 57 (1997) 304-309.

[2] C. Raffel, L. Frederick, J.R. O'Fallon, P. Atherton-Skaff, A. Perry, R.B. Jenkins, C.D. James, Analysis of oncogene and tumor suppressor gene alterations in pediatric malignant astrocytomas reveals reduced survival for patients with PTEN mutations, Clin Cancer Res 5 (1999) 4085-4090.

[3] T. Sung, D.C. Miller, R.L. Hayes, M. Alonso, H. Yee, E.W. Newcomb, Preferential inactivation of the p53 tumor suppressor pathway and lack of EGFR amplification distinguish de novo high grade pediatric astrocytomas from de novo adult astrocytomas, Brain Pathol 10 (2000) 249-259.

[4] M. Nakamura, K. Shimada, E. Ishida, T. Higuchi, H. Nakase, T. Sakaki, N. Konishi, Molecular pathogenesis of pediatric astrocytic tumors, Neuro Oncol 9 (2007) 113-123.
